# Supplementary material for: 2-Year Change in Revised Hammersmith Scale Scores in a Large Cohort of Untreated Paediatric Type 2 and 3 SMA Participants
Source: J Clin Med. 2023 Feb 28;12(5):1920. doi: 10.3390/jcm12051920 (PMC10004034; doi:10.3390/jcm12051920)
Supplement: Supplementary file 1 [file jcm-12-01920-s001.zip › jcm-2203860-supplementary.pdf]

Tables Document:

Grouping		N	RHS Median (IQR)	RHS Range	HFMSE Median (IQR)	HFMSE Range	Age Median (IQR)
All		177	12 (6-34)	0 - 69	18 (7-40)	0 - 66	7.7 (4-10.6)
SMA Type	2	110	7 (4-11)	0 - 27	9 (4-18)	0 - 39	6 (3-9.6)
	3a	58	40 (25-48)	7-67	47 (35-51)	7-63	8.8 (5.3-11.5)
	3b	9	59 (49-64)	31 - 69	60 (53-63)	36 - 66	13.3 (9.4-15.2)
Age Group	<5	60	10 (6-23)	3-52	16 (9-34)	3-53	3.2 (2.2-4)
	5-7	33	15 (7-46)	3-67	22 (10-49)	3-63	6.3 (5.5-7.3)
	8-13	64	16 (5-38)	0 - 65	21 (6-44)	0 - 63	9.8 (9.1-11.8)
	14-18	20	6 (3-27)	0 - 69	7 (3-38)	0 - 66	14.9 (14.6-16.1)
WHO Derived Functional Group	Non-Sitter	22	2 (1-4)	0 - 8	2 (0-4)	0 - 12	10.3 (6.4-12.6)
	Sitter	98	8 (6-15)	2-26	12 (7-20)	2-40	5.8 (3.3-9.6)
	Transitional Group	11	26 (25-29)	18 - 59	36 (34-38)	24 - 61	8.7 (6.3-13.1)
	Walker	46	46 (41-58)	27 - 69	49 (47-58)	35 - 66	8.1 (4.8-10.1)
Ambulation	Non-Ambulant	131	8 (4-15)	0 - 59	11 (5-21)	0 - 61	7.6 (3.8-10.9)
Gender	Male	94	10 (5-24)	0 - 69	14 (7-34)	0 - 66	7.9 (4.5-11.1)
	Female	83	15 (6-40)	0 - 67	21 (9-45)	0 - 63	7.3 (3.6-9.9)
Spinal Surgery	Yes	23	5 (2-10)	0 - 59	6 (2-13)	0 - 61	13.5 (10.2-14.9)
	No	126	14 (6-36)	0 - 69	20 (9-41)	0 - 66	6.3 (3.6-9.5)
RHS Total Score Group	Q1	36	3 (2-4)	0 - 4	3 (1-4)	0 - 8	9.8 (4.9-13.8)
	Q2	38	6 (6-7)	5-9	9 (7-11)	4-16	6.1 (2.5-9.6)
	Q3	38	14 (10-16)	10-18	19 (15-23)	11-29	5.1 (3.7-8.6)
	Q4	34	28 (25-39)	20 - 42	38 (34-46)	27 - 49	9.3 (4.6-10.9)
	Q5	31	56 (46-64)	43 - 69	56 (50-62)	36 - 66	7.9 (5.5-10.1)

Table S1. Patient first visit RHS scores and Ages by SMA Type, Age and Motor Function Level

			6-month				12-month				18-month				24-month			
			M	N	RHS	HFMSE	M	N	RHS	HFMSE	M	N	RHS	HFMSE	M	N	RHS	HFMSE
ALL		Median (IQR)			0 (-1, 1)	0 (-2, 1)			0 (-2, 2)	0 (-3, 2)			0 (-4, 2)	-1 (-5, 1)			0 (-4, 2)	0 (-5, 3)
		P-value	316	124	0.365	0.603	244	112	0.834	0.211	137	82	0.223	0.015	101	63	0.53	0.598
SMA Type	2	Median (IQR)			0 (-1, 1)	0 (-1, 1)			0 (-1, 1)	0 (-2, 1)			0 (-2, 1.5)	0 (-4, 1)			0 (-2.75, 1)	0 (-4.75, 2)
		P-value	188	77	0.419	0.93	144	63	1	0.188	75	43	1	0.117	58	33	0.568	0.672
	3a	Median (IQR)			-1 (-4, 2)	-0.5 (-3, 2)			-1 (-4, 2)	-1 (-4, 2)			-2 (-6, 1.25)	-1.5 (-7, 1.25)			-1 (-9, 4)	-1 (-8, 3)
		P-value	102	39	0.079	0.203	85	44	0.368	0.434	52	34	0.036	0.04	33	27	0.728	0.86
	3b	Median (IQR)			-1 (-2.75, 0.75)	0 (-1, 1)			2 (-2.5, 4)	1 (-1.5, 2.5)			2 (-1.75, 4.5)	0.5 (-1, 2.75)			0.5 (-1.75, 3.75)	0 (-1, 4.5)
		P-value	26	8	0.189	0.824	15	5	0.424	0.424	10	5	0.754	1	10	3	1	1
Age	<5	Median (IQR)			0 (-0.25, 2)	0 (-1, 2)			1 (0, 4)	1 (0, 4)			3 (0, 5)	3 (0, 6)			2 (1, 6)	3 (1, 8)
		P-value	100	44	0.022	0.014	80	37	0	0	39	28	0.001	0.006	29	17	0	0.001
	5-7	Median (IQR)			0 (-1, 1)	0 (-1, 2)			0 (-2, 2)	0 (-3, 2)			-1.5 (-4, 0.25)	-1 (-4, 1)			-1.5 (-4, 1.5)	-2 (-4, 2)
		P-value	66	33	1	0.488	48	30	0.875	1	24	16	0.115	0.263	22	17	0.078	0.664
	8-13	Median (IQR)			-1 (-2, 1)	-1 (-2.75, 0.75)			-1 (-4, 0)	-2 (-5, 0)			-2 (-5.5, 0)	-4 (-7, 0)			-2 (-8, 0)	-4 (-9, 0)
		P-value	118	53	0.01	0.001	97	47	0	0	63	34	0	0	43	30	0.005	0.001
	14-18	Median (IQR)			-1 (-1.25, 1)	0 (-2, 0)			-1 (-2.5, 0.5)	0 (-2, 0.5)			0 (-0.5, 0.5)	0 (-1, 1)			0 (-0.5, 2)	0 (-1, 3)
		P-value	32	14	0.185	0.359	19	14	0.302	0.581	11	8	1	0.727	7	2		1
WHO Derived Functional Group	Non-Sitter	Median (IQR)			0 (-0.75, 0.75)	0 (0, 1)			0 (-1, 1.5)	0 (-1, 0.5)			0 (-1, 0)	0 (0, 0)			-0.5 (-1, 0.25)	0 (-0.25, 0.25)
		P-value	14	11	1	0.219	15	12	1	0.754	9	7			4	4		
	Sitter	Median (IQR)			0 (-1, 1)	0 (-2, 1)			0 (-1, 1)	0 (-2, 1.75)			0 (-3, 2)	-1 (-4, 1)			0 (-2, 2)	0 (-5, 3)
		P-value	188	77	1	0.261	138	63	0.702	0.259	68	40	0.892	0.111	53	30	1	1
	Transitional	Median (IQR)			-1 (-3.25, 1)	-1 (-4, 1)			-3 (-5, -2)	-3 (-6, -1)			-6 (-7, -2.5)	-6 (-9, -3)			-7 (-7, -4.5)	-4 (-7.5, -4)
		P-value	24	11	0.383	0.383	21	13	0	0.001	14	9	0	0	7	5	0.016	0.016
	Walker	Median (IQR)			-0.5 (-3, 2)	0 (-2, 1.75)			1 (-3.75, 4)	1 (-3, 3)			0 (-3.75, 4)	0 (-4, 3)			0 (-9, 4)	0 (-5, 3)
		P-value	90	33	0.26	0.567	70	34	0.321	0.215	46	30	1	0.878	37	27	1	1
Ambulation	Non-Ambulant	Median (IQR)			0 (-1, 1)	0 (-2, 1)			0 (-2, 1)	0 (-3, 1)			0 (-3.5, 1)	-1 (-5, 1)			0 (-3, 1)	0 (-5, 2)
		P-value	226	95	0.813	0.27	174	82	0.312	0.015	91	55	0.125	0.005	64	38	0.419	0.504

Table S2. Up to 2-year median change in the RHS and HFMSE broken down by SMA type, age and motor function group

Age		<5				5-7				8-13				14-18			
SMA Type		6m	12m	18m	24m	6m	12m	18m	24m	6m	12m	18m	24m	6m	12m	18m	24m
2	M	89	67	29	22	39	24	12	11	51	46	30	24	9	7	4	1
	N	37	28	19	12	20	15	8	7	27	23	15	16	5	6	3	1
	RHS Median (IQR)	0 (0, 2)	1 (0, 2)	2 (0, 5)	1 (1, 4.5)	0 (-1, 0)	-0.5 (-2, 0.25)	-2 (-4, 0)	-2 (-4, -1)	0 (-1, 1)	-1 (-2, 0)	-1 (-3.75, 0)	-1.5 (-4.25, 0)	0 (-1, 1)	0 (-1, 0)	0 (0, 0)	
	P-value	0.01	0	0.002	0.003	0.405	0.238	0.18	0.021	0.487	0.003	0.023	0.019				
	HFMSE Median (IQR)	0 (0, 2)	1 (0, 4)	2 (0, 5)	2 (1, 5.75)	0 (-2, 1)	-1 (-3.25, 0.25)	-2 (-5, 0.25)	-4 (-4.5, -2)	0 (-2, 0)	-1 (-3.75, 0)	-4 (-6, 0)	-3 (-8, 0)	0 (0, 0)	0 (-0.5, 0)	-0.5 (-1, 0)	
	P-value	0.006	0.002	0.015	0.001	0.442	0.052	0.344	0.065	0.024	0	0	0.004				
3a	M	10	13	10	7	26	22	12	11	58	44	28	15	8	6	2	0
	N	6	9	9	5	12	14	8	10	23	21	16	12	4	5	2	0
	RHS Median (IQR)	-0.5 (-3.25, 2.25)	6 (4, 8)	4 (-1.5, 5.75)	6 (4.5, 9)	0.5 (-3.5, 3.75)	0.5 (-2.75, 2.75)	-1 (-4, 1.5)	-1 (-4.5, 3.5)	-1 (-3.75, 1)	-3 (-5, 0.25)	-3 (-7, 0)	-9 (-12.5, 0)	-1.5 (-3.25, 1)	-2.5 (-3, -0.5)	-3	
	P-value	0.727	0.003	0.344		0.678	0.824	0.549	1	0.03	0.004	0.003	0.118				
	HFMSE Median (IQR)	0 (-1.75, 1.75)	3 (1, 4)	3.5 (-2.25, 6)	6 (1, 8)	1 (-0.75, 3)	1.5 (-2.75, 3.75)	-0.5 (-3, 2)	2 (-1.5, 3.5)	-1 (-4, 0.75)	-3 (-6.25, 0)	-4.5 (-9.25, -0.75)	-6 (-10, -2)	-2 (-4, 1)	-2 (-2, -0.5)	-1.5	
	P-value	1	0.006	0.344		0.064	0.189	0.754	0.344	0.007	0.001	0.001	0.035				
3b	M	1	0	0	0	1	2	0	0	9	7	5	4	15	6	5	6
	N	1	0	0	0	1	1	0	0	3	3	3	2	5	3	3	1
	RHS Median (IQR)						2			-3 (-8, 0)	1 (-4, 3.5)	3 (-2, 3)	0 (-5.25, 5.75)	-1 (-1.5, 0.5)	1 (-2.75, 4.75)	1 (-1, 5)	0.5 (-0.75, 2.5)
	P-value																
	HFMSE Median (IQR)						3			1 (-2, 2)	1 (-1.5, 2)	1 (-1, 3)	1.5 (-1.25, 3.5)	0 (-1, 1)	0.5 (-2.25, 1.75)	0 (-1, 2)	-0.5 (-1, 3.75)
	P-value																

Table S3. Up to 2-year median change in the RHS and HFMSE cross-tabulated by SMA type and age

Age		<5				5-7				8-13				14-18			
Functional Type		6m	12m	18m	24m	6m	12m	18m	24m	6m	12m	18m	24m	6m	12m	18m	24m
Non-Sitter	M	3	3	1	0	1	0	1	0	9	10	6	4	1	2	1	0
	N	3	2	1	0	1	0	1	0	6	8	4	4	1	2	1	0
	RHS Median (IQR)	0 (-0.5, 1.5)	5 (3.5, 5.5)							0 (0, 1)	-1 (-1, 0)	-0.5 (-1, 0)	-0.5 (-1, 0.25)	-1 (-1, -1)	0		
	P-value										0.289						
	HFMSE Median (IQR)	1 (0.5, 1.5)	7 (6, 10)							0 (0, 1)	-1 (-1, 0)	0 (0, 0)	0 (-0.25, 0.25)	0 (0, 0)	0		
	P-value										0.125						
Sitter	M	81	61	26	22	38	23	8	9	56	45	29	21	13	9	5	1
	N	33	27	17	12	20	15	7	7	28	22	14	13	7	7	4	1
	RHS Median (IQR)	0 (0, 2)	1 (0, 2)	2.5 (1, 5)	1.5 (1, 5.75)	0 (-1, 0.75)	0 (-1, 1.5)	-1 (-3.25, 0)	-1 (-2, 0)	-1 (-1.25, 0.25)	-1 (-3, 0)	-2 (-4, 0)	-2 (-5, 0)	0 (-1, 1)	-1 (-1, 0)	0 (0, 0)	
	P-value	0.014	p<0.001	p<0.001	p<0.001	0.832	1		0.289	0.032	0.002	0.023	0.013	1	0.453		
	HFMSE Median (IQR)	0 (0, 3)	1 (0, 3)	3 (0, 6.5)	2.5 (1, 6)	0 (-1, 1)	-1 (-2, 1)	-0.5 (-3.25, 0.25)	-2 (-4, 1)	-1 (-3, 0)	-2 (-5, 0)	-4 (-7, -2)	-5 (-8, -1)	0 (-2, 0)	-1 (-2, 0)	-1 (-1, 0)	
	P-value	0.018	0.002	0.004	p<0.001	0.69	0.664	0.687	0.508	p<0.001	p<0.001	p<0.001	0.001	0.219	0.453		
Transitional Group	M	5	4	2	1	5	6	7	4	10	9	5	2	4	2	0	0
	N	2	2	1	1	2	3	3	2	5	7	5	2	3	2	0	0
	RHS Median (IQR)	1 (0, 2)	-1.5 (-2.25, -0.25)	-1.5	-1 (-1, -1)	-1 (-3, 0)	-3 (-4.5, -2.25)	-4 (-6, -3)	-7 (-7.5, -6.25)	-2.5 (-3.75, -0.25)	-3 (-6, -2)	-7 (-7, -7)	-6	-0.5 (-2.75, 1)	-3.5		
	P-value										0.004						
	HFMSE Median (IQR)	1 (0, 1)	-1.5 (-3, 0.25)	-2.5	-2 (-2, -2)	1 (-4, 3)	-3.5 (-5.5, -1.5)	-5 (-6, -3)	-4 (-5.5, -4)	-2.5 (-4.75, -0.25)	-5 (-9, -3)	-11 (-11, -9)	-8.5	-2 (-3.75, 0.25)	-1		
	P-value										0.004						
Walker	M	11	12	10	6	22	19	8	9	43	33	23	16	14	6	5	6
	N	7	9	9	5	10	13	5	8	17	14	15	13	4	3	3	1
	RHS Median (IQR)	0 (-2.5, 3)	5 (3.5, 8)	4 (-1.5, 5.75)	6 (3.75, 7.5)	1 (-3.5, 3)	1 (-3, 2.5)	0.5 (-1, 3.75)	-1 (-3, 4)	-1 (-3, 1)	0 (-5, 2)	-2 (-5.5, 1.5)	-7.5 (-11.25, 3.25)	-1 (-1.75, 0)	1 (-2.75, 4.75)	1 (-1, 5)	0.5 (-0.75, 2.5)
	P-value	1	0.006	0.344		0.503	0.815		1	0.256	0.711	0.263	0.454				
	HFMSE Median (IQR)	0 (-1.5, 1.5)	3 (1, 4.5)	3.5 (-2.25, 6)	5.5 (0, 8)	1 (-0.75, 2)	2 (-2.5, 3.5)	0.5 (-1, 5.25)	2 (0, 4)	0 (-2, 1.5)	-1 (-3, 2)	-1 (-5.5, 1)	-4 (-10, 1.25)	0 (-0.75, 1)	0.5 (-2.25, 1.75)	0 (-1, 2)	-0.5 (-1, 3.75)
	P-value	1	0.012	0.344		0.078	0.238		0.289	0.743	0.473	0.189	0.302				

Table S4. Up to 2-year median change in the RHS and HFMSE cross-tabulated by WHO-Derived Functional type and age

Age		<5				5-7				8-13				14-18			
RHS Baseline Total		6m	12m	18m	24m	6m	12m	18m	24m	6m	12m	18m	24m	6m	12m	18m	24m
Q1: 0-4	M	17	20	8	9	8	3	1	0	19	19	11	9	8	6	4	1
	N	9	10	6	3	3	1	1	0	11	11	6	8	5	5	3	1
	RHS Median (IQR)	1 (0, 2)	1 (0.75, 1)	1 (0.75, 3)	1 (1, 2)	0 (-1, 0)	0 (-0.5, 0)			0 (0, 0.5)	0 (-1, 0)	0 (0, 0.5)	0 (-1, 0)	0 (-1, 1)	0 (-0.75, 0)	0 (0, 0)	0 (0, 0)
	P-value	0.002	0.001	0.031						1	0.388						
	HFMSE Median (IQR)	1 (0, 2)	0.5 (0, 3)	1 (0, 3.5)	2 (0, 2)	0 (-0.25, 0.5)	1 (1, 1.5)			0 (0, 0)	-1 (-1, 0)	0 (-0.5, 0)	0 (-1, 0)	0 (0, 0)	0 (0, 0)	-0.5 (-1, 0)	1 (1, 1)
	P-value	0.065	0.092							1	0.012						
Q2: 5-9	M	39	25	10	5	15	7	2	3	16	12	8	5	1	2	0	0
	N	18	11	6	4	11	5	2	3	10	7	4	2	1	2	0	0
	RHS Median (IQR)	0 (0, 2)	2 (0, 5)	5.5 (1.5, 7.75)	8 (7, 9)	0 (-0.5, 1)	2 (0, 2)	1	0 (-0.5, 1)	0 (-1, 0.25)	-1 (-2, -0.75)	-2 (-3.25, -0.5)	-2 (-3, -1)		0.5		
	P-value	0.076	0.004	0.004		0.754				0.549	0.065						
	HFMSE Median (IQR)	1 (0, 3)	2 (0, 8)	6 (1.5, 10.25)	9 (9, 11)	0 (-1, 0.5)	0 (-1.5, 2)	1	1 (-0.5, 2)	-1 (-2.5, 0)	-2.5 (-4, -1)	-4.5 (-6, -4)	-6 (-8, -4)		0		
	P-value	0.043	0.004	0.002		0.754				0.065	0.012						
Q3: 10-18	M	27	18	9	7	14	12	6	5	20	16	12	11	1	1	2	0
	N	14	11	8	5	8	10	5	3	11	9	8	7	1	1	2	0
	RHS Median (IQR)	0 (-1, 1)	0 (-1, 1.75)	2 (-1, 3)	1 (0.5, 2)	0 (-0.75, 0)	0 (-1, 0.25)	-2.5 (-3.75, -0.5)	-1 (-3, -1)	-1 (-2.25, 1)	-2.5 (-5, -0.75)	-3 (-4.75, -0.75)	-5 (-7, -1.5)			-3	
	P-value	0.824	1	0.508		1	1			0.332	0.035	0.021	0.065				
	HFMSE Median (IQR)	0 (-1.5, 1)	0.5 (-0.75, 3)	1 (-3, 3)	3 (1, 5)	0 (-2, 1)	-1 (-2.5, 0.25)	-2 (-3.75, -0.25)	-3 (-4, -2)	-1.5 (-3, 0.5)	-3 (-7.25, -1.75)	-4.5 (-9.5, -2.75)	-6 (-11.5, -3.5)			-1.5	
	P-value	0.824	0.424	0.727		1	0.227			0.064	0.007	0.012	0.065				
Q4: 19-42	M	12	14	8	6	10	12	8	6	30	25	15	9	10	6	1	0
	N	7	8	6	4	5	8	4	4	16	16	10	7	4	4	1	0
	RHS Median (IQR)	0.5 (-0.25, 2)	4 (0.25, 7.5)	3.5 (-1.25, 6)	7 (6, 9.5)	-1.5 (-3.75, 0.75)	-2.5 (-5.25, -1.25)	-4 (-6, -2)	-5.5 (-7, -2.5)	-2 (-3.75, -0.25)	-4 (-6, -1)	-6 (-10.5, -4.5)	-9 (-10, -5)	0.5 (-2.5, 1)	-1.5 (-3, 3)		
	P-value	0.508	0.092	0.727			0.146			0.004	0.001	0.001	0.18				
	HFMSE Median (IQR)	0.5 (0, 1.25)	1.5 (-0.5, 3.75)	3 (-2.25, 6)	4.5 (0, 7.5)	1 (-3, 1.75)	-3.5 (-4.25, 1.25)	-4 (-5.5, -2.25)	-4 (-4.75, -4)	-2 (-4, 0)	-5 (-9, -1)	-9 (-11, -5.5)	-6 (-10, -2)	-0.5 (-3.5, 0.75)	-1 (-2, 0.75)		
	P-value	0.289	0.18	0.727			0.388			0.019	p<0.001	0.002	0.07				
Q5: 43-69	M	5	3	4	2	19	14	7	8	33	25	17	9	12	4	4	6
	N	4	3	4	2	9	9	4	7	12	10	11	9	4	2	2	1
	RHS Median (IQR)	-1 (-4, 3)	4 (3, 10.5)	-1 (-6.5, 4.25)	-6	1 (-3, 2.5)	1 (-3.5, 2)	0 (-1, 2)	-1.5 (-3.75, 4.5)	0 (-2, 2)	0 (-4, 3)	0 (-3, 2)	-5 (-15, 3)	-1 (-2, -1)	-2.5 (-3.5, -0.25)	0 (-1.75, 2)	0.5 (-0.75, 2.5)
	P-value					0.629	0.581		0.727	0.711	1	0.791	1				
	HFMSE Median (IQR)	-1 (-2, -1)	1 (0.5, 5)	-1 (-6.75, 4.75)	0	1 (-2, 2.5)	2.5 (-1.5, 3.75)	0 (-1, 3.5)	2 (-0.75, 5)	0 (-2, 2)	0 (-2, 3)	-1 (-5, 1)	-3 (-9, 2)	0 (-2.25, 0.25)	-1.5 (-3.5, 1.25)	-0.5 (-1.5, 1)	-0.5 (-1, 3.75)
	P-value					0.238	0.267		0.453	0.851	1	0.454	1				

Table S5. Up to 2-year median change in the RHS and HFMSE cross-tabulated by baseline RHS group and age

		HFMSE Total Score	
		0	1
RHS Total	0	6	2
	1	7	0
	2	6	0

Table S6. Count of patients scoring 0 on the RHS and the HFMSE
